# Supplementary material for: Screening and Characterization of TAT-Fused Nanobodies Targeting Bovine Viral Diarrhea Virus NS3/NS5A for Antiviral Application
Source: Biomolecules. 2025 Nov 13;15(11):1593. doi: 10.3390/biom15111593 (PMC12650317; doi:10.3390/biom15111593)
Supplement: Supplementary file 1 [file biomolecules-15-01593-s001.zip › Supplementary File/Supplementary Material Explanation.docx]

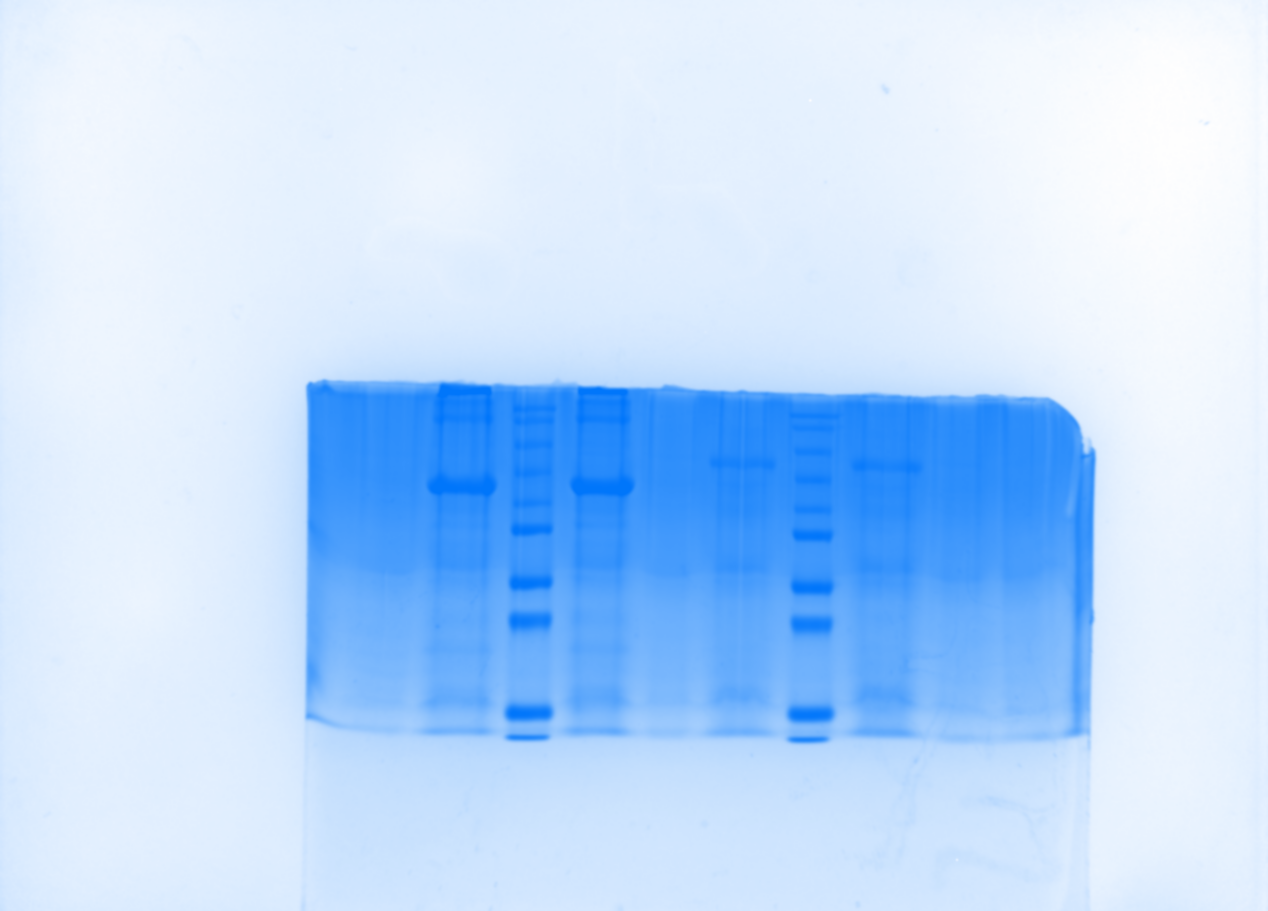


1 M 2 3 M 4

**Figure S1.** Original Images of Figure 1A and 1B (SDS-PAGE Results of NS3 and NS5A Antigen Protein Expression) M: Protein Marker; Lane 1, 2: Purified NS3 protein; Lane 3, 4: Purified NS5A protein.


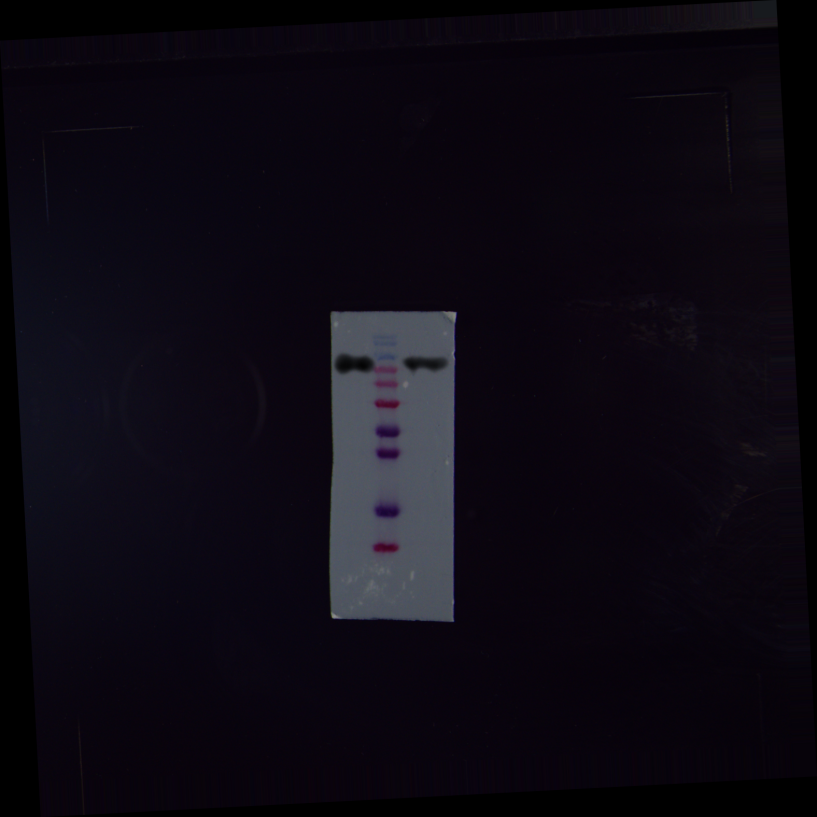


1 M 2

**Figure S2.** Original Image of Figure 1C (Western Blotting Validation Results of NS3 Antigen Protein Expression) M: Protein Marker; Lane 1, 2: Purified NS3 protein validated by Western blotting using an anti-His antibody.


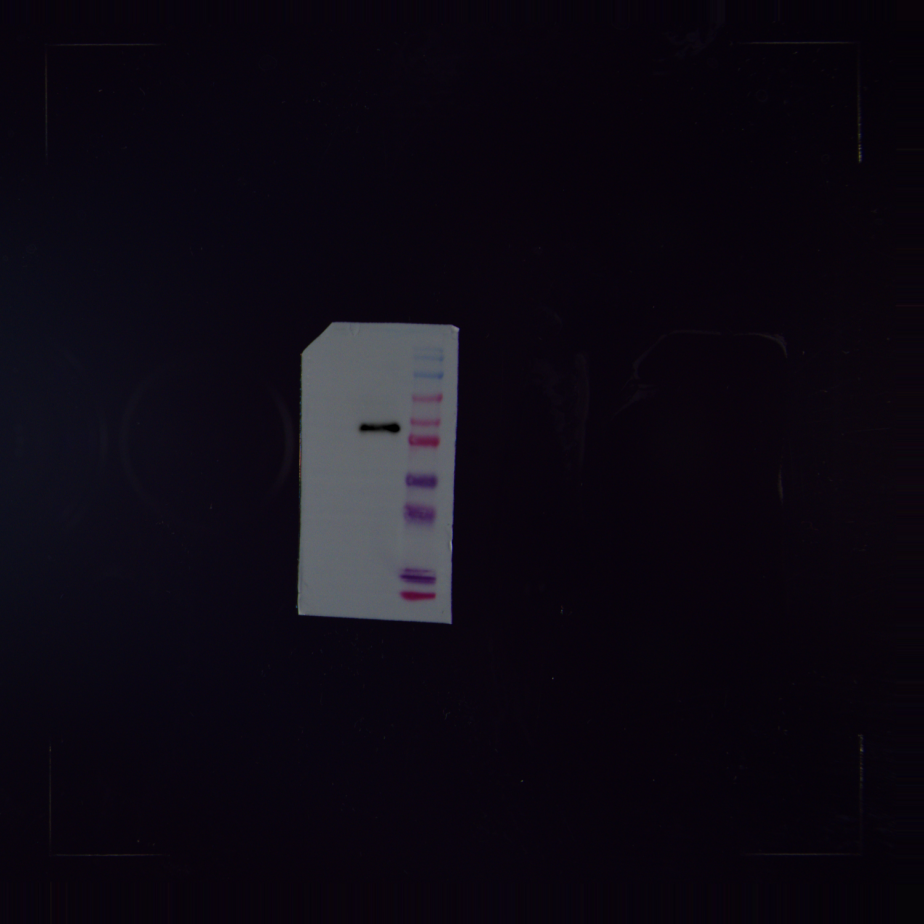


1 M

**Figure S3.** Original Image of Figure 1D (Western Blotting Validation Results of NS5A Antigen Protein Expression) M: Protein Marker; Lane 1: Purified NS5A protein validated by Western blotting using an anti-His antibody.


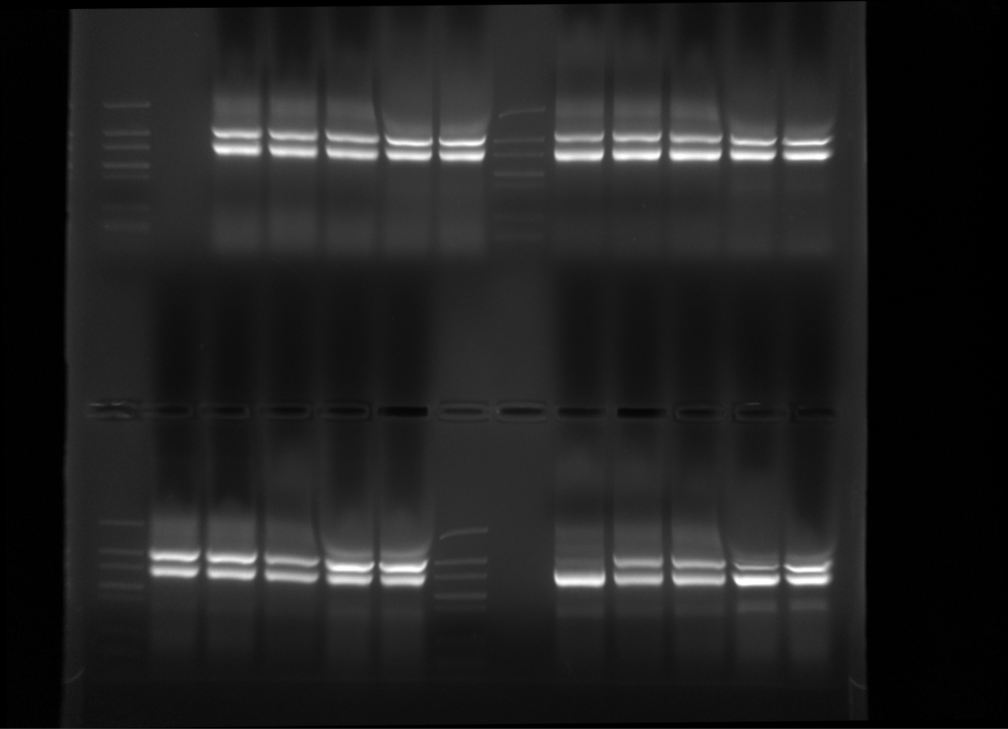


M 1 2

**Figure S4.** Original Image of Figure 2C (A Target Band of Approximately 700 bp Obtained via One Round of PCR Amplification) M: DNA Marker; Lane 1, 2: 1st-round PCR products (~700 bp).


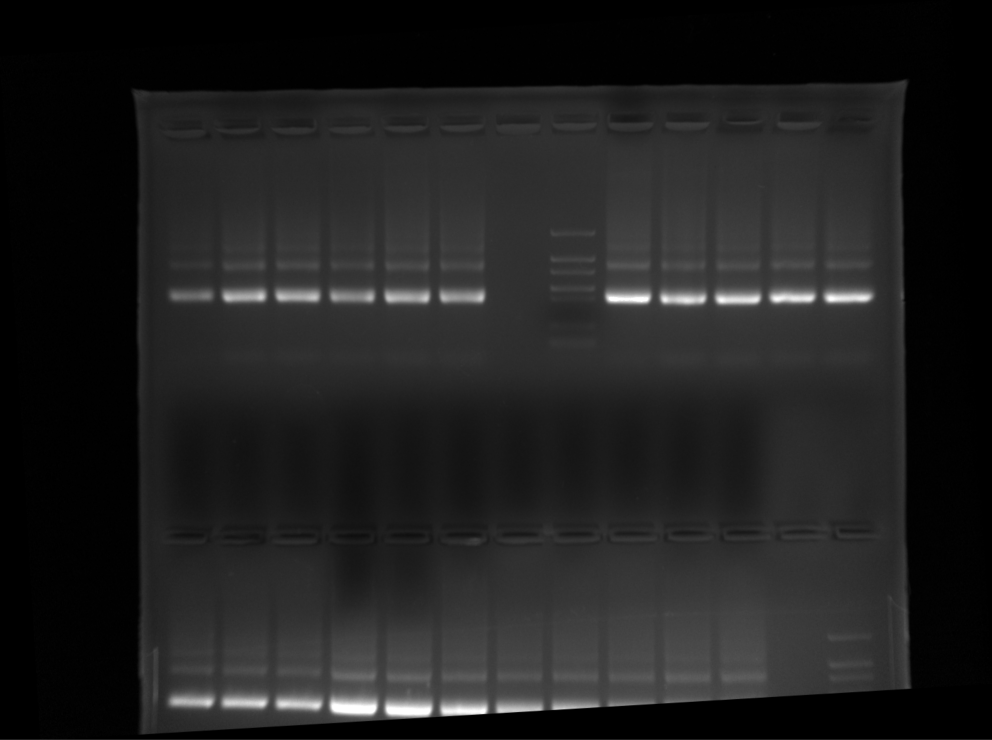


M 1 2

**Figure S5.** Original Image of Figure 2D (A Target Band of Approximately 400 bp Obtained via Two Rounds of PCR Amplification) M: DNA Marker; Lane 1, 2: 2nd-round PCR products (~400 bp).


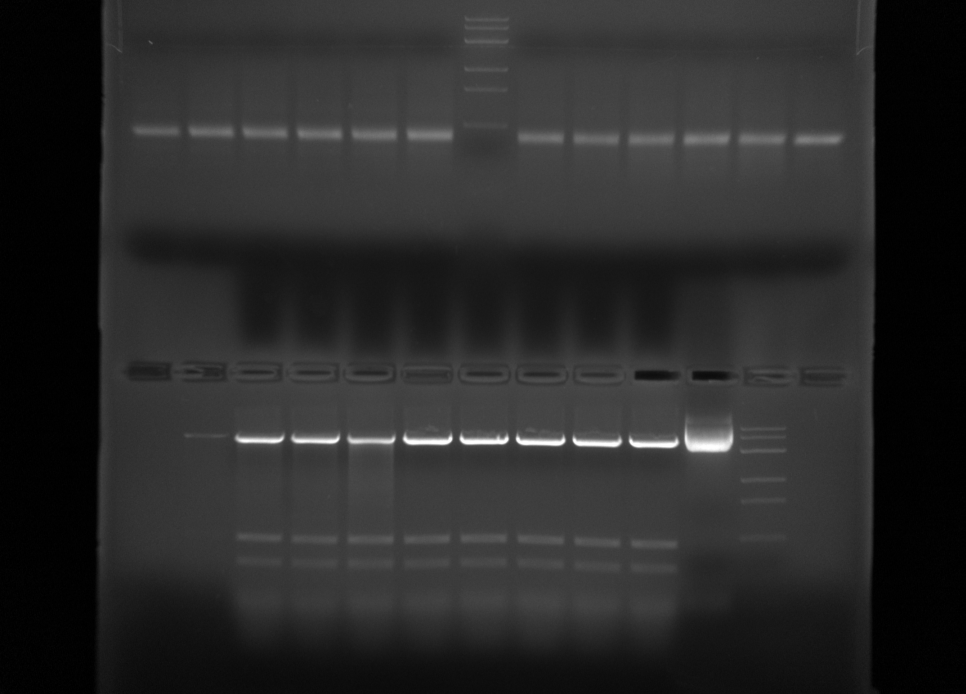


3 2 1 M

**Figure S6.** Original Image of Figure 2E (Double Enzyme Digestion Identification of pCANTAB-5E Plasmid) Lane 1: Empty pCANTAB-5E plasmid; Lane 2, 3: pCANTAB-5E plasmid after double enzyme digestion.


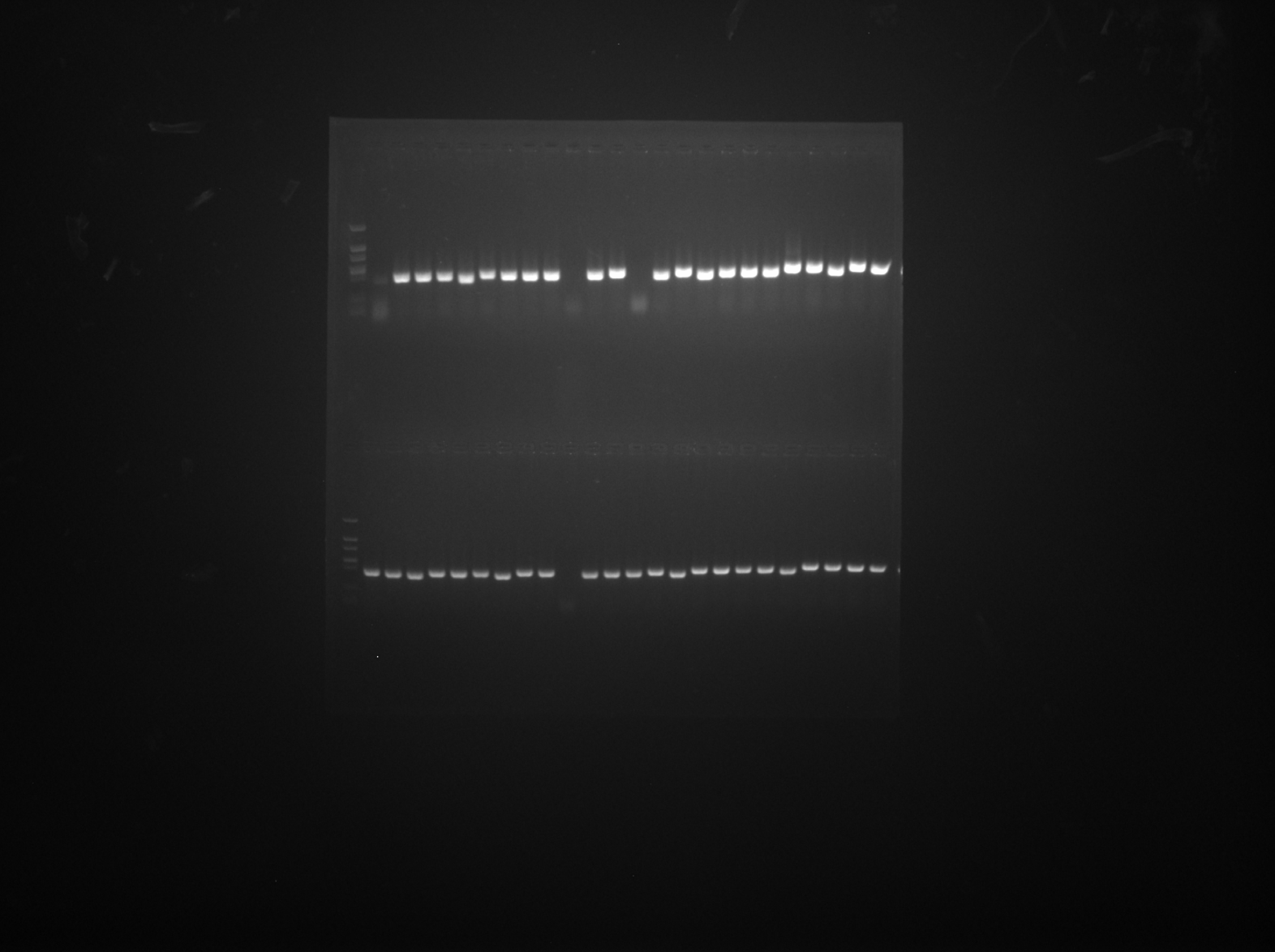


**Figure S7.** Original Image of Figure 2G (PCR Detection of 48 Positive Clones) The correct insertion rate was 91% (44/48).

A B C


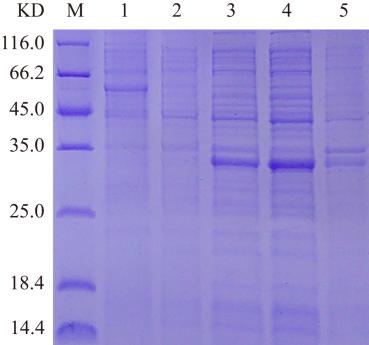

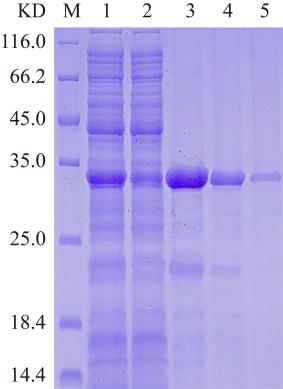

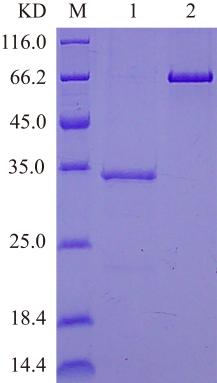


**Figure S8.** Original Image of Figure 4A (Expression and Purification of TAT-Nb7)

A: M: Protein Marker; Lane 1: Induced pET28a-sumo vector (empty vector); Lane 2: Uninduced sample; Lane 3: Induced sample; Lane 4: Supernatant after induced cell lysis; Lane 5: Precipitate after induced cell lysis.

B: M: Protein Marker; Lane 1: Processed sample after cell lysis; Lane 2: Flow-through fraction; Lane 3–5: Eluted fractions.

C: M: Protein Marker; Lane 1: Purified TAT-Nb7 sample; Lane 2: BSA (0.5 mg/mL).

A B C


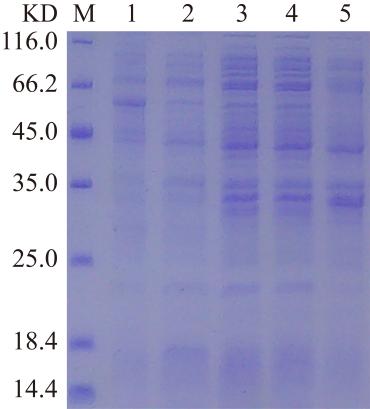

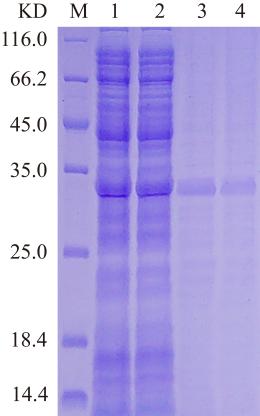

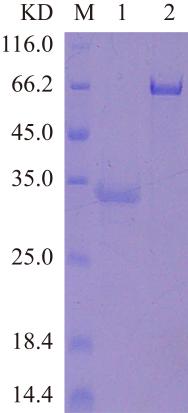


**Figure S9.** Original Image of Figure 4B (Expression and Purification of TAT-Nb23)

A: M: Protein Marker; Lane 1: Induced pET28a-sumo vector (empty vector); Lane 2: Uninduced sample; Lane 3: Induced sample; Lane 4: Supernatant after induced cell lysis; Lane 5: Precipitate after induced cell lysis.

B: M: Protein Marker; Lane 1: Processed sample after cell lysis; Lane 2: Flow-through fraction; Lane 3, 4: Eluted fractions.

C: M: Protein Marker; Lane 1: Purified TAT-Nb23 sample; Lane 2: BSA (0.5 mg/mL).


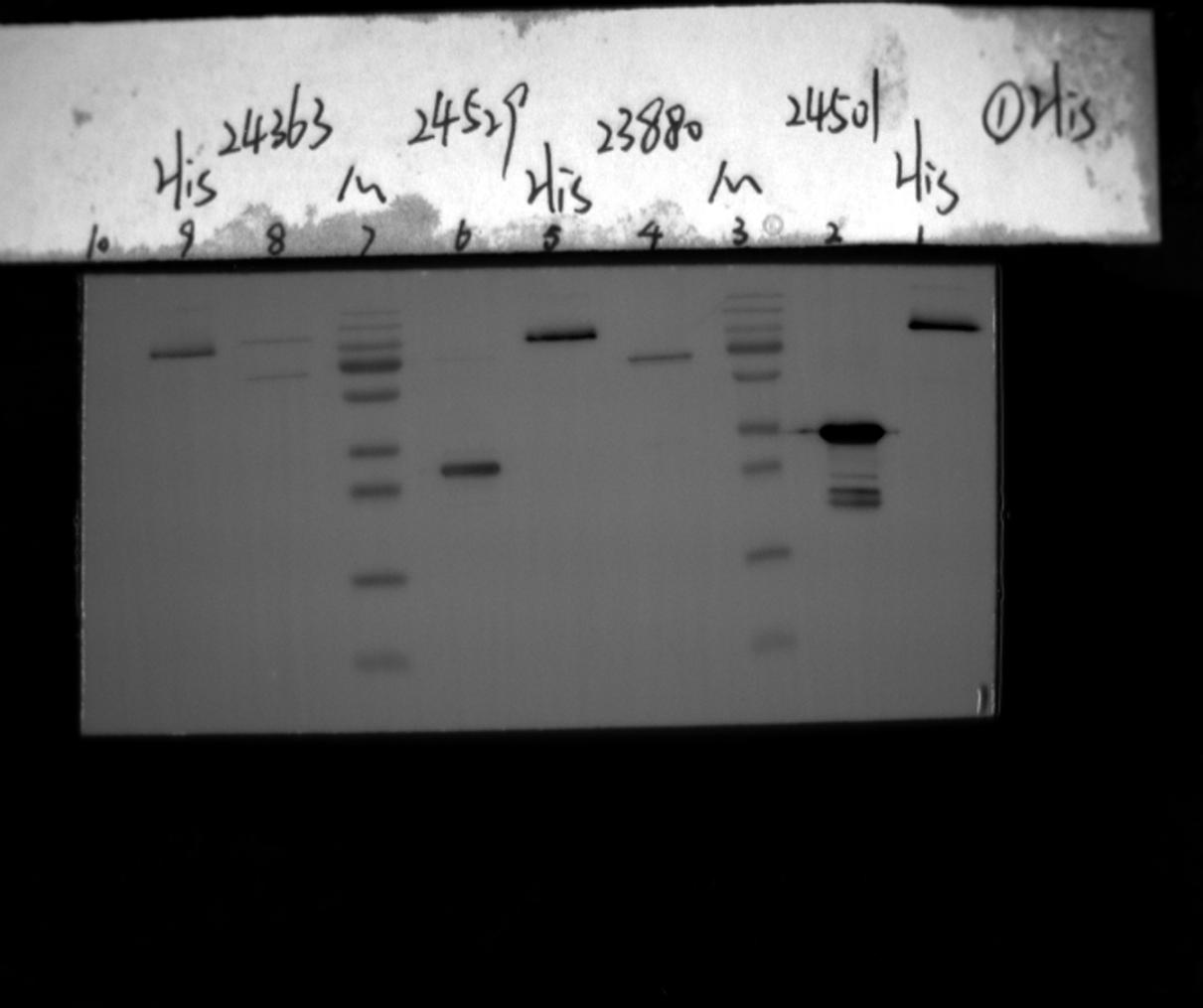


M 1 2

**Figure S10.** Original Image of Figure 4C (Western Blot Identification and Analysis of TAT-Nb7 Protein)M: Protein Marker; Lane 1: Purified TAT-Nb7 sample; Lane 2: Multitag Protein.


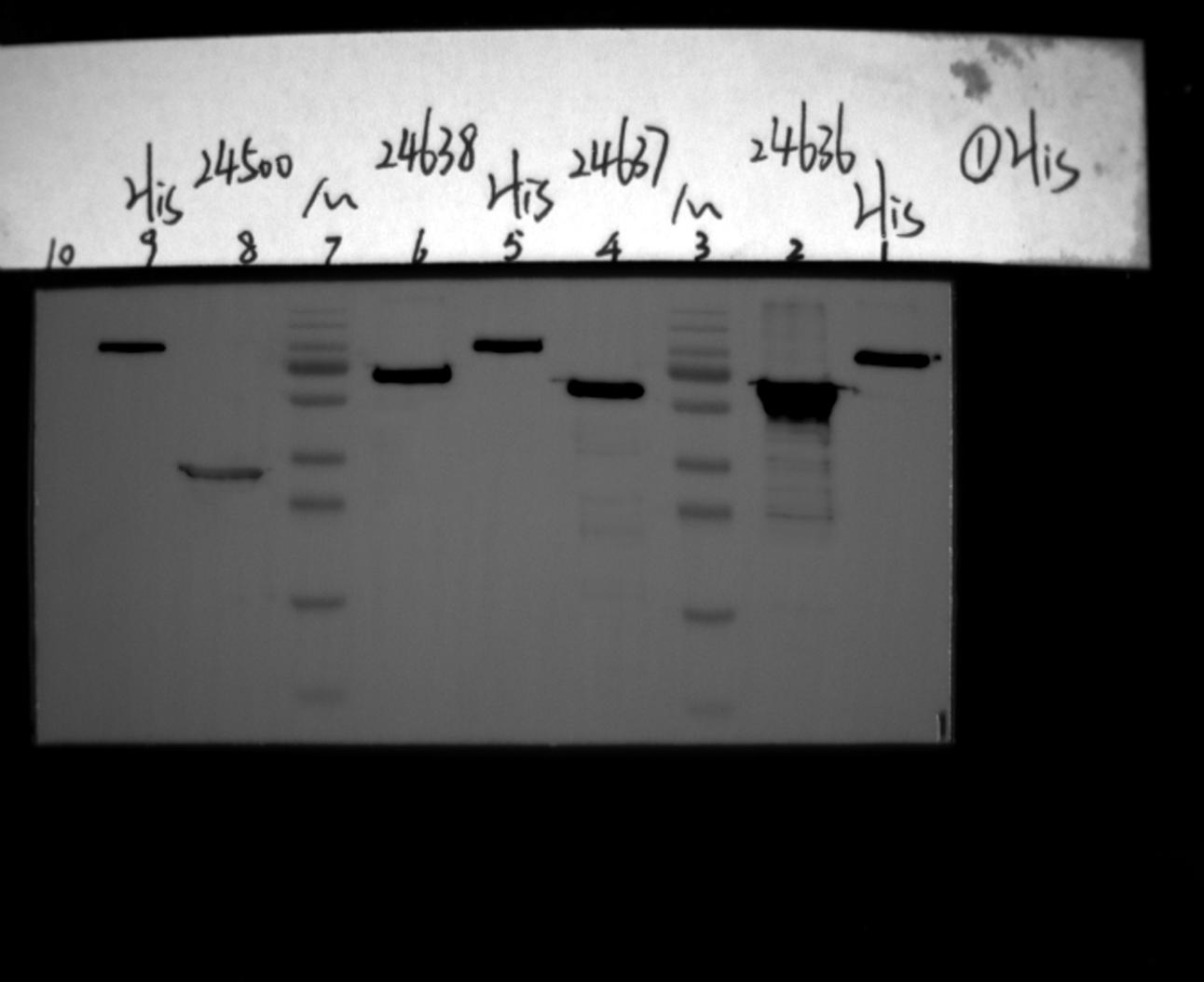


2 1 M

**Figure S11.** Original Image of Figure 4D (Western Blot Identification and Analysis of TAT-Nb23 Protein) M: Protein Marker; Lane 1: Purified TAT-Nb23 sample; Lane 2: Multitag Protein.


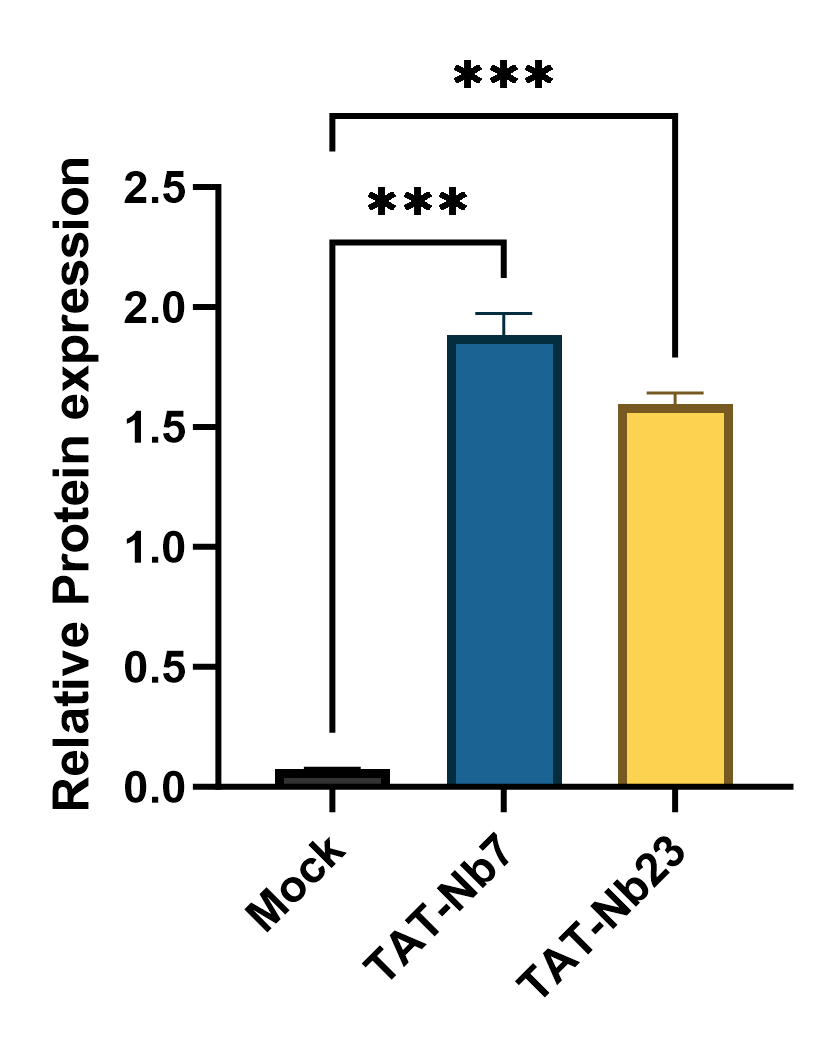


**Figure S12.** (Figure 5B. Western Blot Validation of the Transmembrane Efficiency of TAT-Nb7 and TAT-Nb23) Band gray value analysis was performed using ImageJ v1.49 software, and graphing was conducted with Graphpad Prism 8 software.


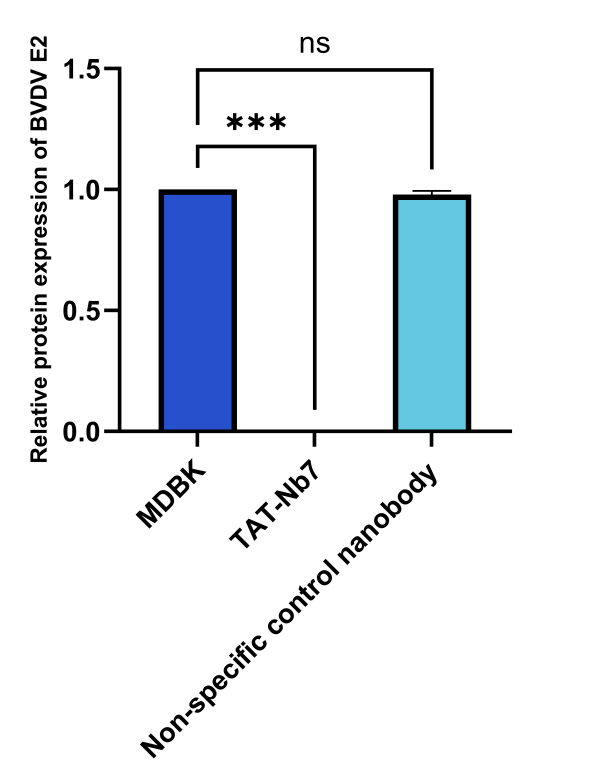


**Figure S13.** (Figure 6C. Detection of the effect of TAT-Nb7 on BVDV replication at 48 hours post viral challenge using Western blot.) Band gray value analysis was performed using Image J software, and graphing was conducted with Graphpad Prism 8 software.


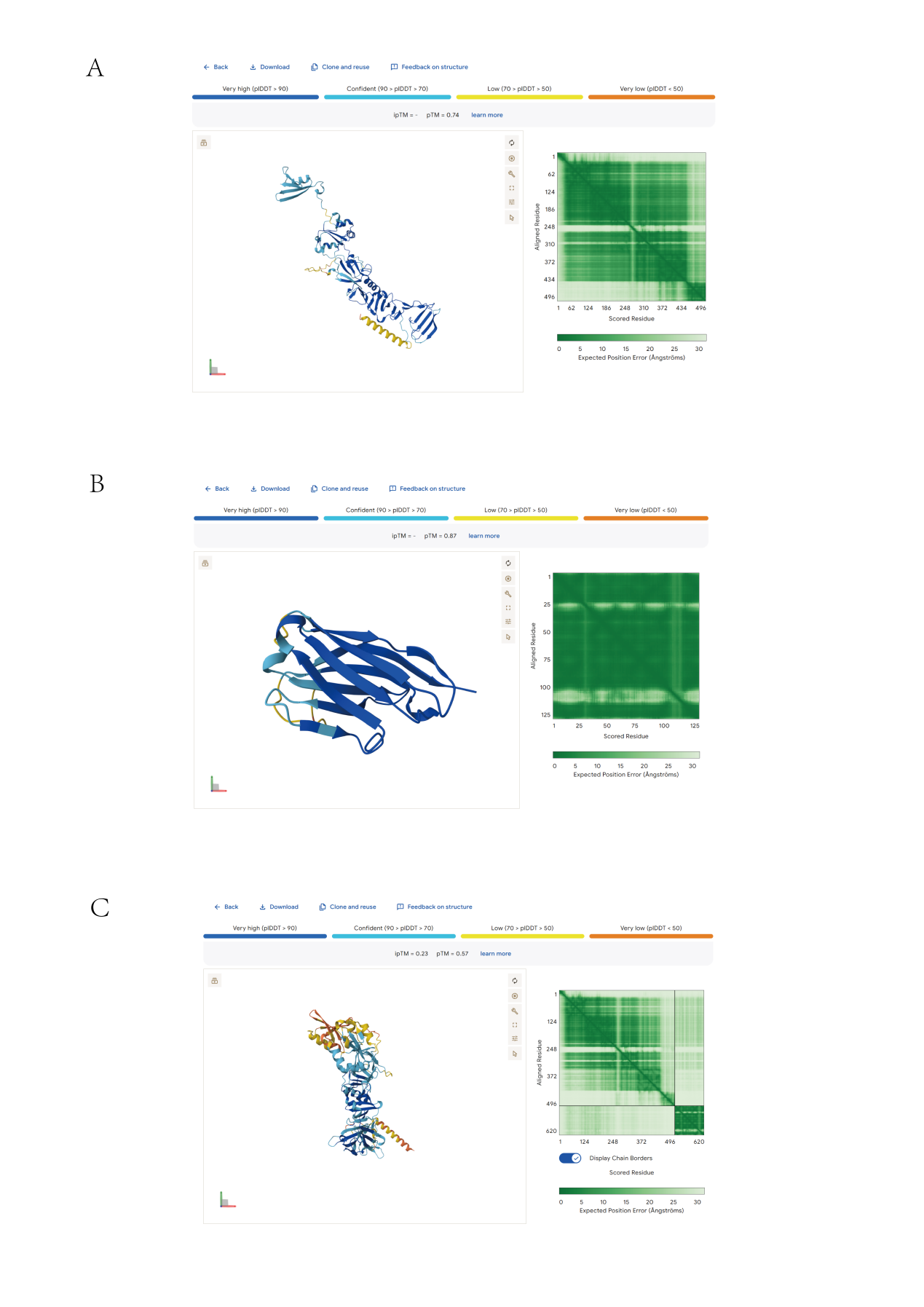


**Figure S14：**(Molecular docking model of TAT-Nb7 and NS5A. ) Figure A: Structure prediction of NS5A antigen protein, Figure B: Structure prediction of TAT-Nb7 protein, Figure C: Stability prediction of protein docking structure.pLDDT (predicted local distance difference test) is an indicator used by AlphaFold to evaluate the confidence of each residue prediction. The higher the score (>90 for very high, 70-90 for confidence, 50-70 for low,<50 for very low), the more reliable the structure. The pLDDT values of the NS5A antigen protein and TAT-Nb7 protein are mostly distributed in the 70-90 range, indicating a confident predicted structure. The consistency between the predicted contact and the experiment also reflects the rationality of the structure.The ipTM value indicates that the interactive interface is reliable; The PAE plot shows that the root mean square error of predicting atomic positions between key residues is within a reasonable range, reflecting the stability of complex structures. The darker the color, the more likely the residue pairs are to come into contact in the experiment.

Table S1. Hydrogen bonds between NS5A and TAT-Nb7

| No. | NS5A | Dist | TAT-Nb7 |
| --- | --- | --- | --- |
| 1 | A:ARG 44 [NH2] | 3.71 | B:SER 32 [OG] |
| 2 | A:TYR 157 [OH] | 2.84 | B:GLN 1 [OE1] |
| 3 | A:LYS 109 [NZ] | 2.44 | B:ASP 113 [OD1] |
| 4 | A:ALA 112 [N] | 3.36 | B:MET 101 [O] |
| 5 | A:LYS 155 [NZ] | 2.64 | B:ASP 59 [OD1] |
| 6 | A:TYR 157 [OH] | 2.88 | B:GLY 102 [O] |
| 7 | A:PRO 46 [O] | 3.46 | B:LEU 29 [N] |
| 8 | A:LYS 155 [O] | 3.11 | B:SER 104 [OG] |
| 9 | A:LYS 155 [O] | 3.60 | B:MET 106 [N] |
| 10 | A:GLU 107 [O] | 3.42 | B:TYR 114 [OH] |

Table S2. Salt bridges between NS5A and TAT-Nb7

| No. | NS5A | Dist | TAT-Nb7 |
| --- | --- | --- | --- |
| 1 | A:LYS 109 [NZ] | 2.44 | B:ASP 113 [OD1] |
| 2 | A:LYS 109 [NZ] | 3.49 | B:ASP 113 [OD2] |
| 3 | A:LYS 135 [NZ] | 2.80 | B:ASP 55 [OD2] |
| 4 | A:LYS 155 [NZ] | 2.64 | B:ASP 59 [OD1] |
| 5 | A:LYS 155 [NZ] | 3.63 | B:ASP 59 [OD2] |
| 6 | A:ASP 102 [OD2] | 2.89 | B:GLN 1 [N] |

Table S3. PISA Interface List

| NS5A | | | | TAT-Nb7 | | | | | Protein docking | |
| --- | --- | --- | --- | --- | --- | --- | --- | --- | --- | --- |
| Range | ^i^Nat | ^i^Nres | Surface Å^2^ | | Range | ^i^Nat | ^i^Nres | Surface Å^2^ | interface  area, Å^2^ | Δ^i^G  kcal/mol |
| 1 | 109 | 28 | 29729 | | 2 | 96 | 26 | 6461 | 981.2 | -4.1 |

^i^Nat indicates the number of interfacing atoms in the corresponding structure. ^i^Nres indicates the number of interfacing residues in the corresponding structure. Surface Å^2^ is the total solvent accessible surface area in square Ångstroms. Interface area in Å^2^, calculated as difference in total accessible surface areas of isolated and interfacing structures divided by two. Δ^i^G indicates the solvation free energy gain upon formation of the interface, in kcal/M. The value is calculated as difference in total solvation energies of isolated and interfacing structures. Negative Δ^i^G corresponds to hydrophobic interfaces, or positive protein affinity.
