# Supplementary figures and images for: Screening and Characterization of TAT-Fused Nanobodies Targeting Bovine Viral Diarrhea Virus NS3/NS5A for Antiviral Application
Source: Biomolecules. 2025 Nov 13;15(11):1593. doi: 10.3390/biom15111593 (PMC12650317; doi:10.3390/biom15111593)

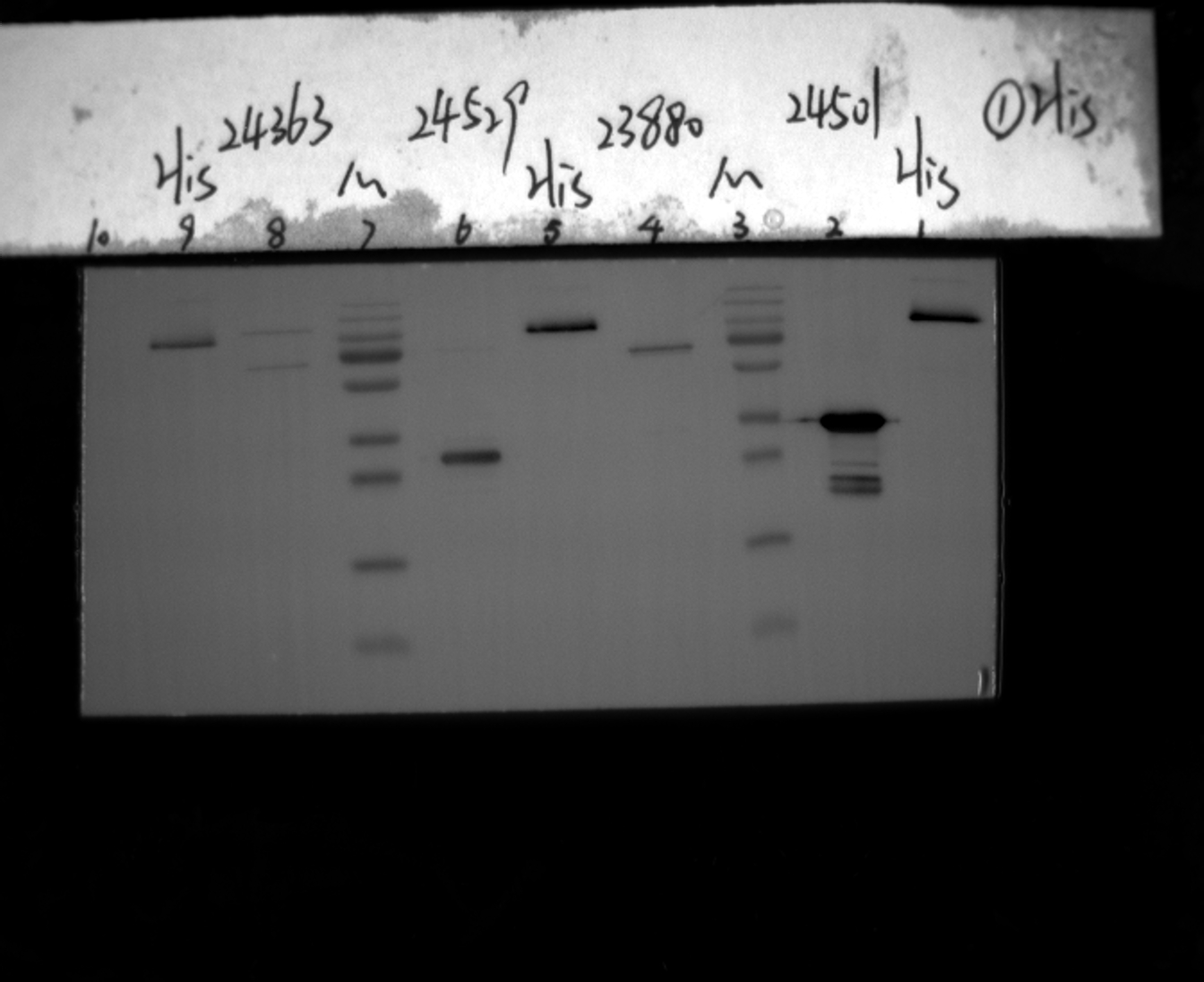

Supplement: Supplementary file 1 [file biomolecules-15-01593-s001.zip › Supplementary File/Figure S10. Original Image of Figure 4C (Western Blot Identification and Analysis of TAT-Nb17 Protein).jpg]

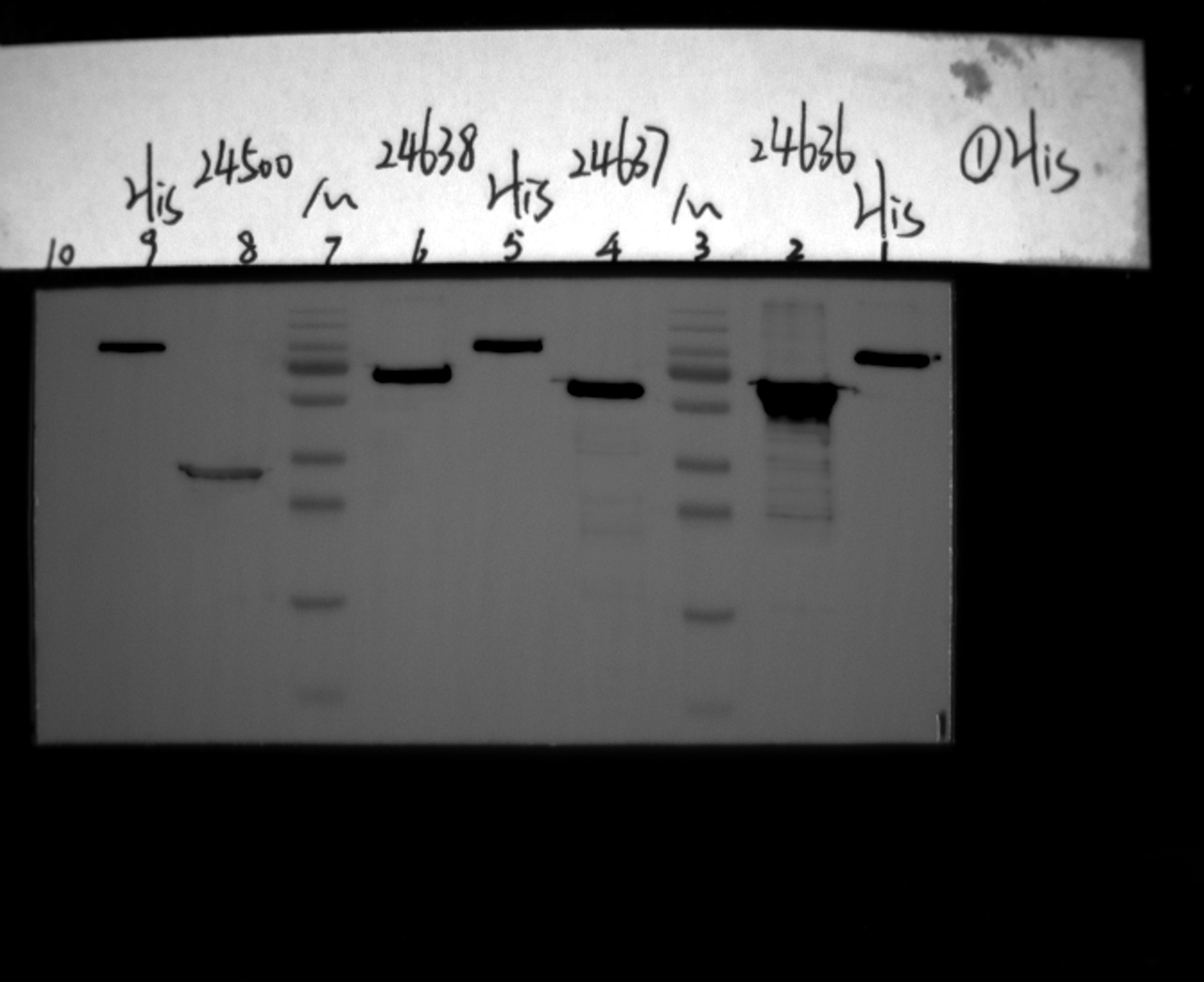

Supplement: Supplementary file 1 [file biomolecules-15-01593-s001.zip › Supplementary File/Figure S11. Original Image of Figure 4D (Western Blot Identification and Analysis of TAT-Nb23 Protein) .jpg]

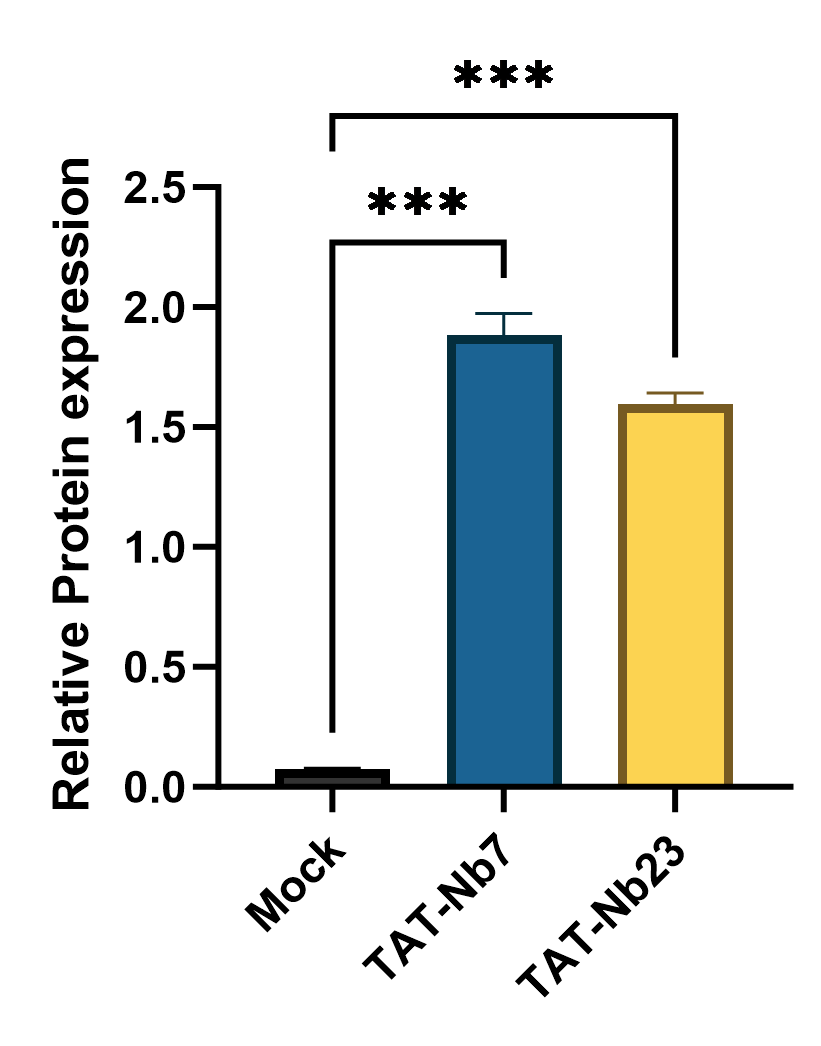

Supplement: Supplementary file 1 [file biomolecules-15-01593-s001.zip › Supplementary File/Figure S12.png]

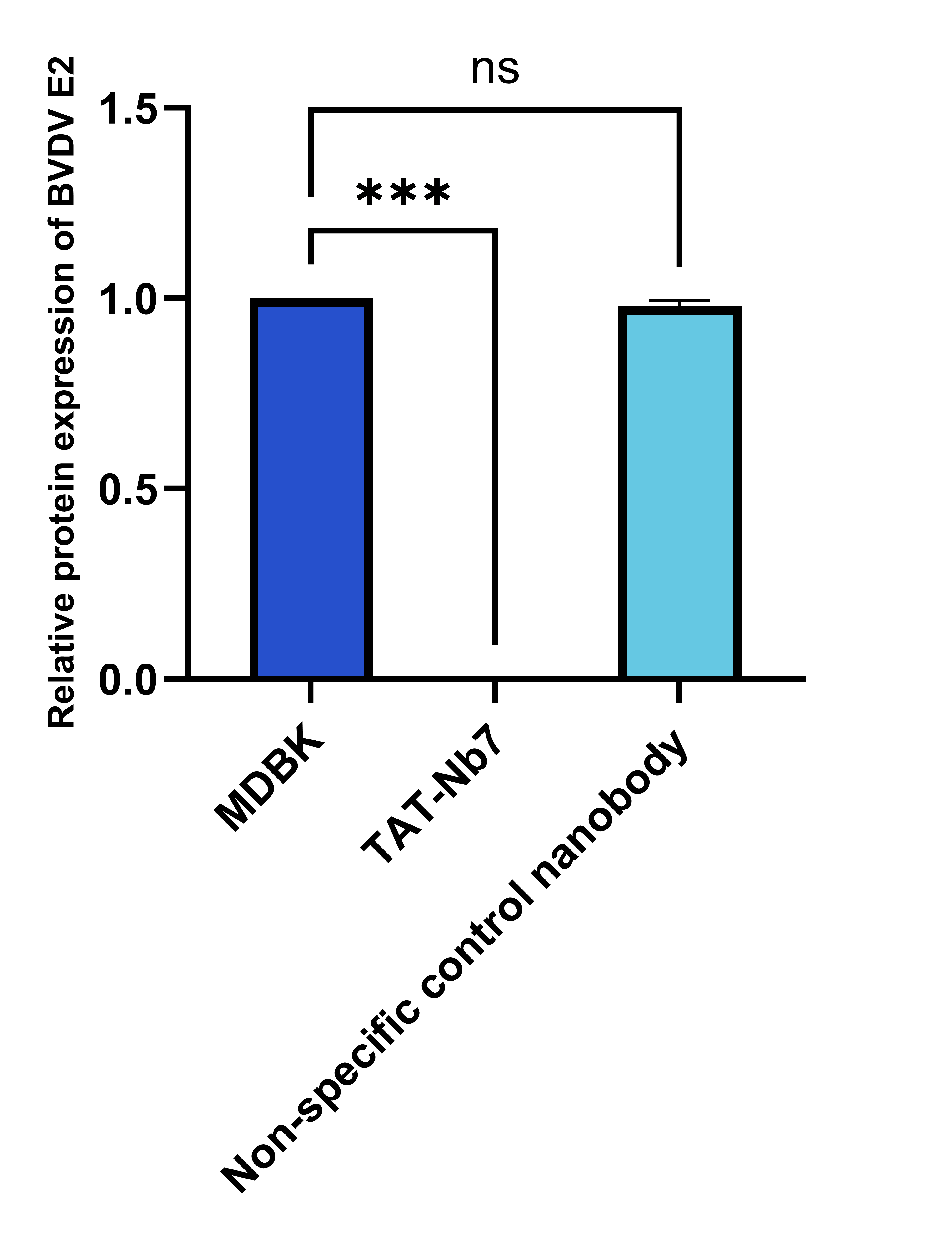

Supplement: Supplementary file 1 [file biomolecules-15-01593-s001.zip › Supplementary File/Figure S13.png]

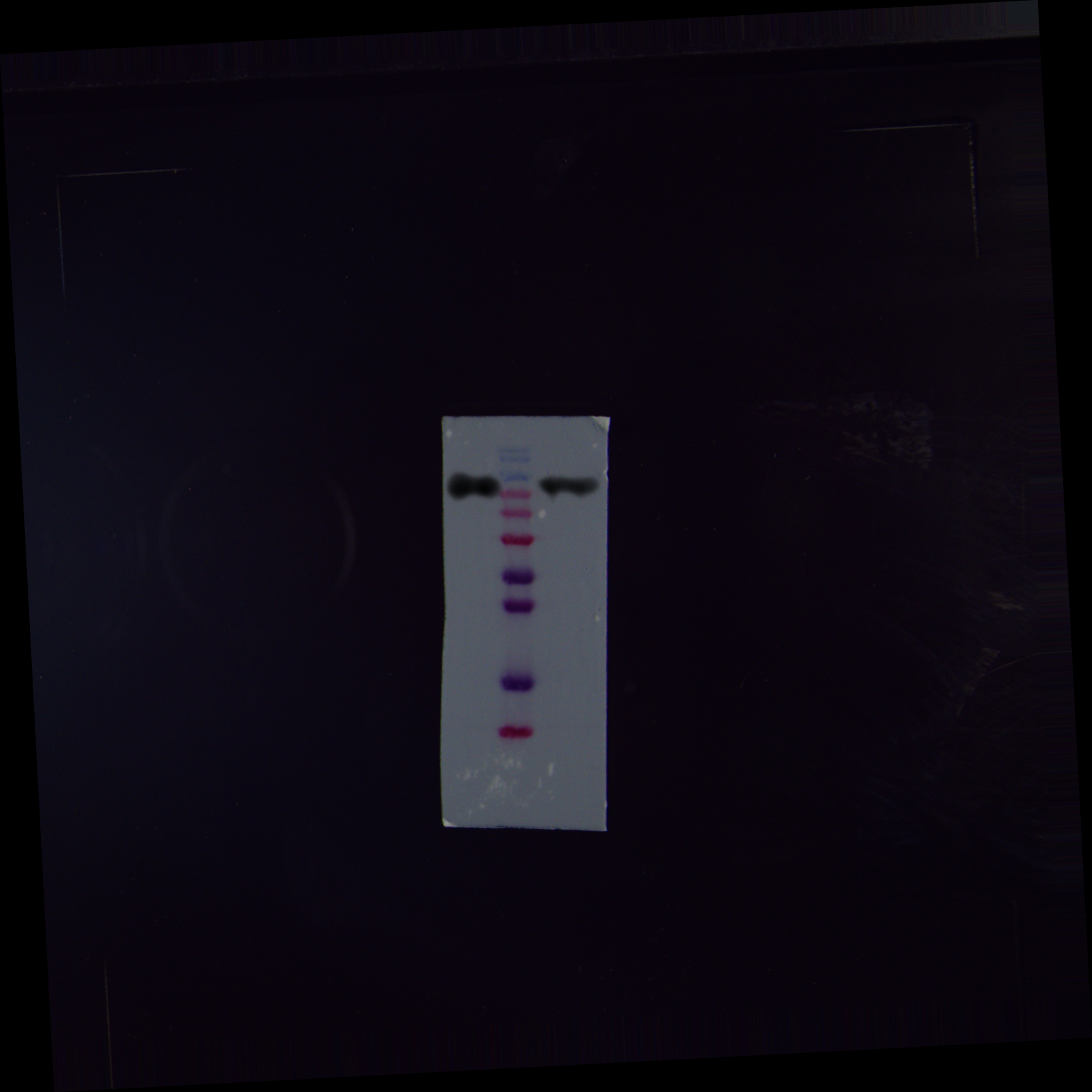

Supplement: Supplementary file 1 [file biomolecules-15-01593-s001.zip › Supplementary File/Figure S2ú║Original image of Figure 1C (Western blotting validation results of NS3 antigen protein expression) .tiff]

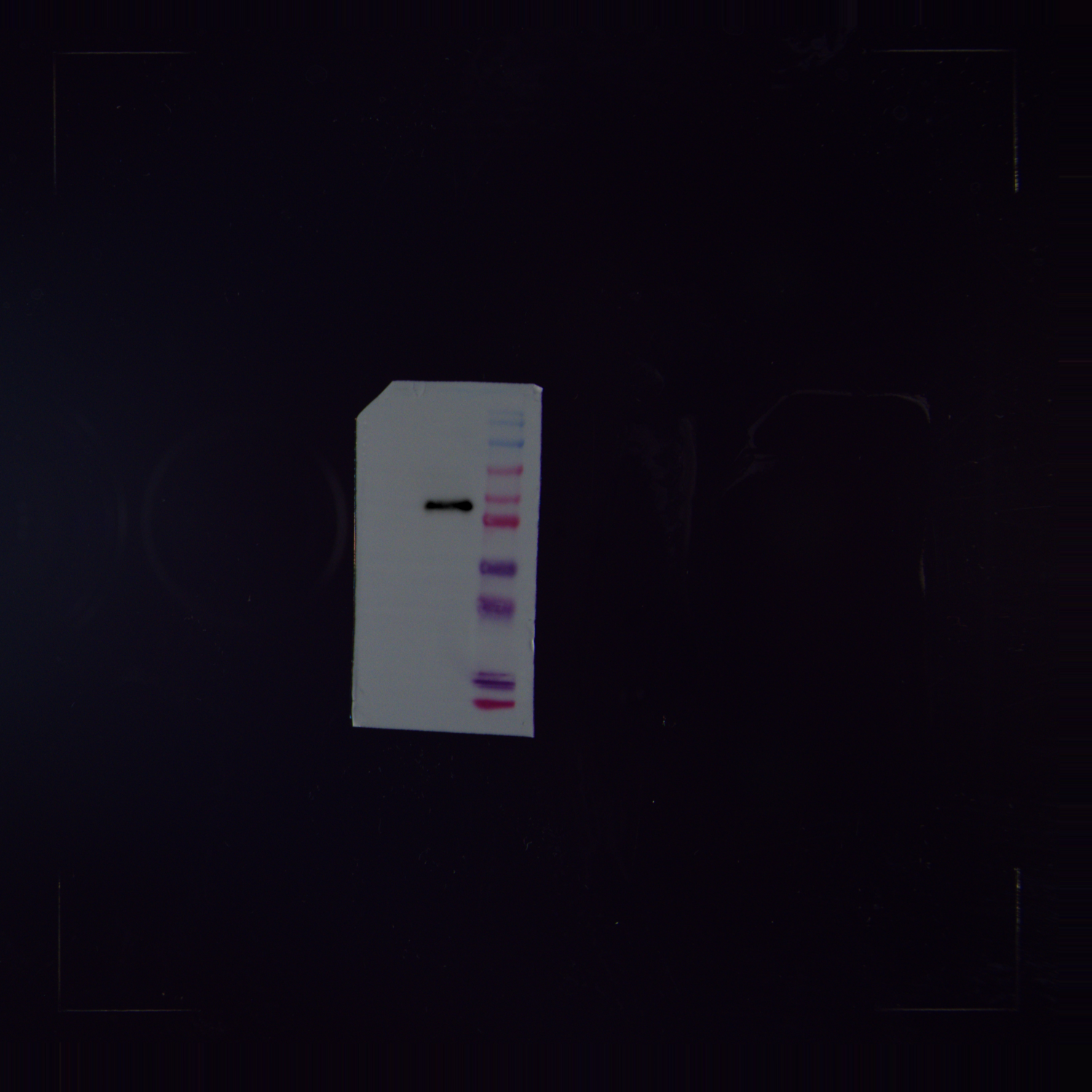

Supplement: Supplementary file 1 [file biomolecules-15-01593-s001.zip › Supplementary File/Figure S3ú║Original image of Figure 1D ú¿Western blotting validation results of NS5A antigen protein expressionú⌐.tiff]

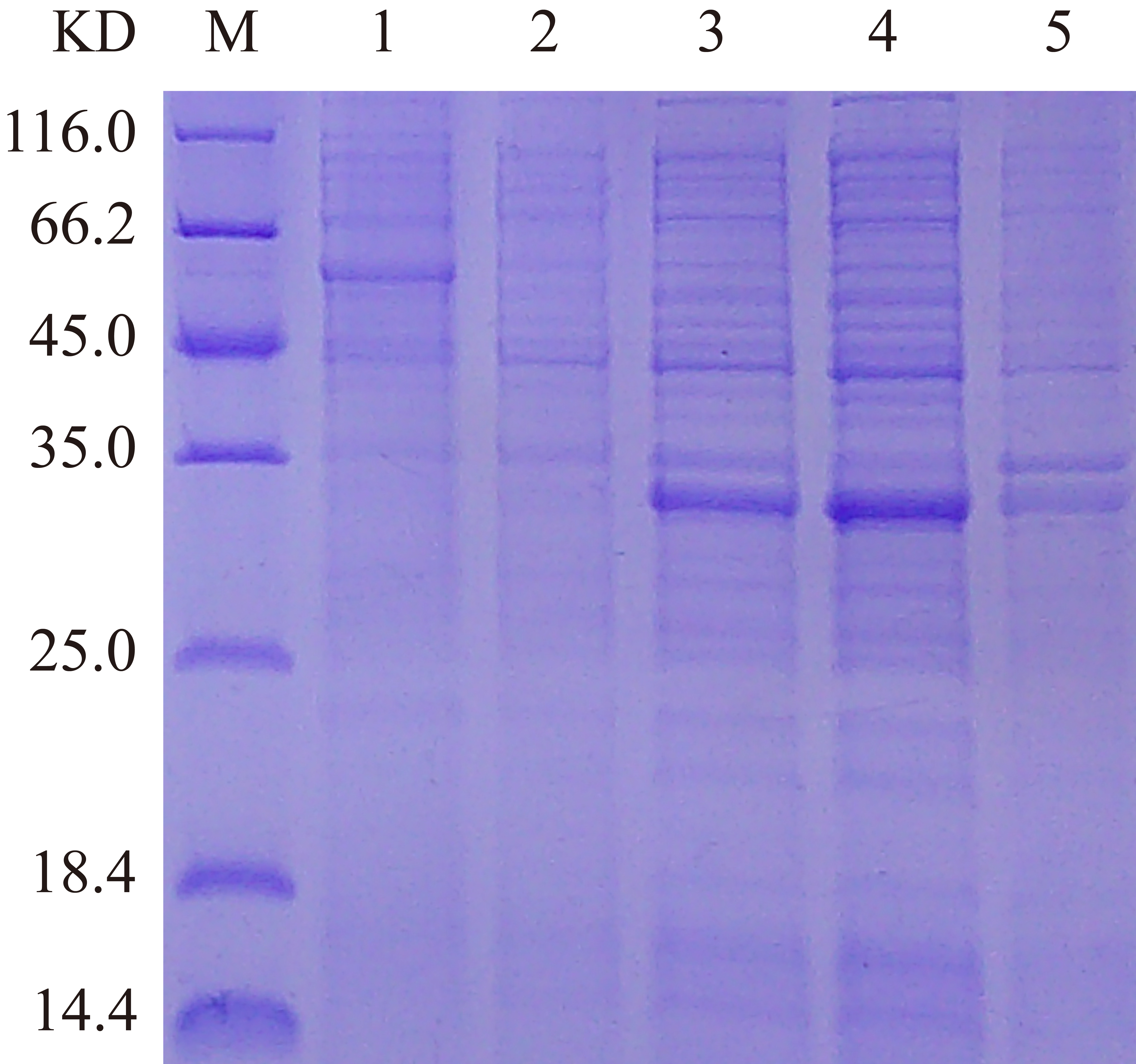

Supplement: Supplementary file 1 [file biomolecules-15-01593-s001.zip › Supplementary File/Figure S8-ú¿Aú⌐.tiff]

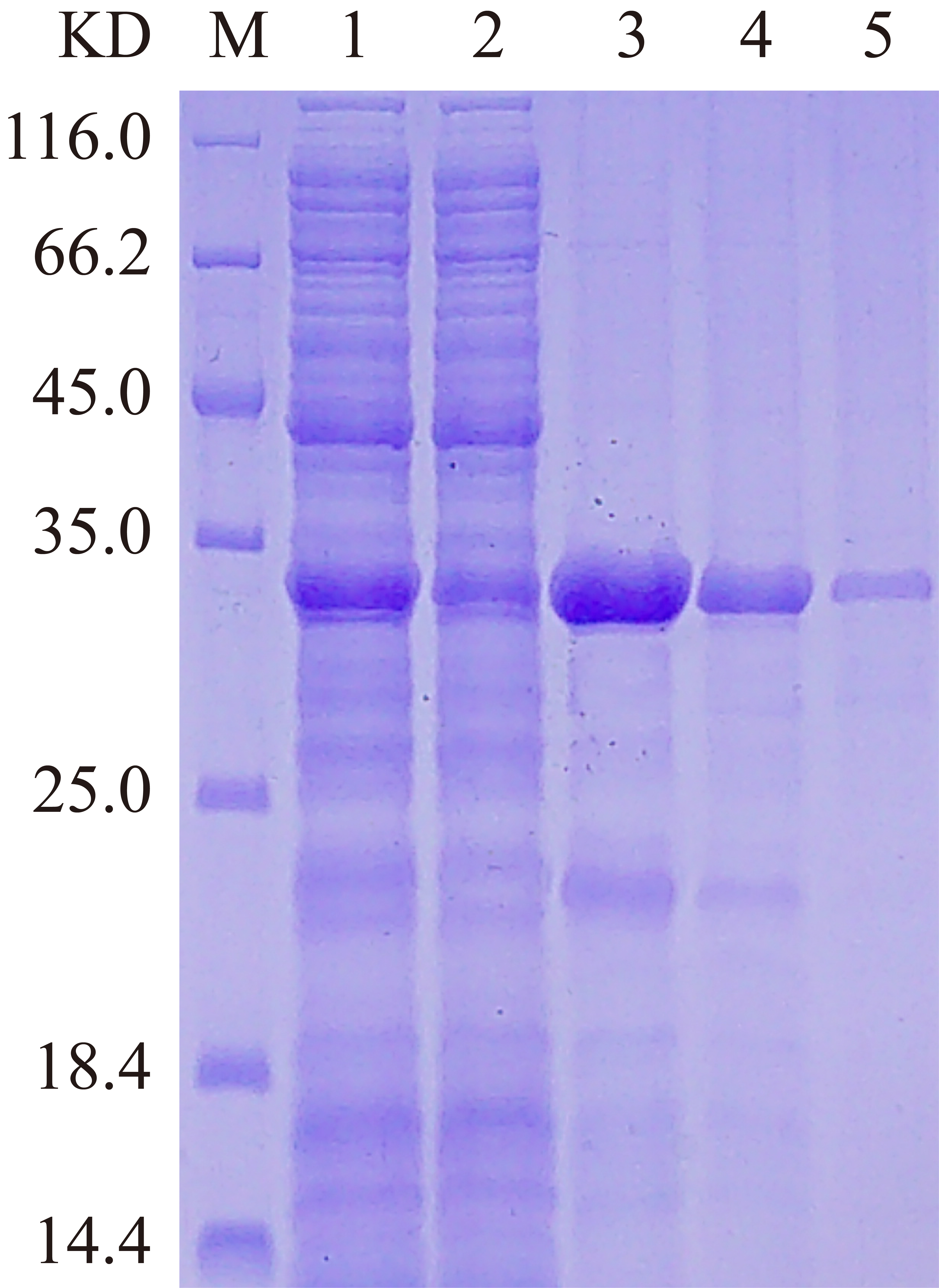

Supplement: Supplementary file 1 [file biomolecules-15-01593-s001.zip › Supplementary File/Figure S8-ú¿Bú⌐.tiff]

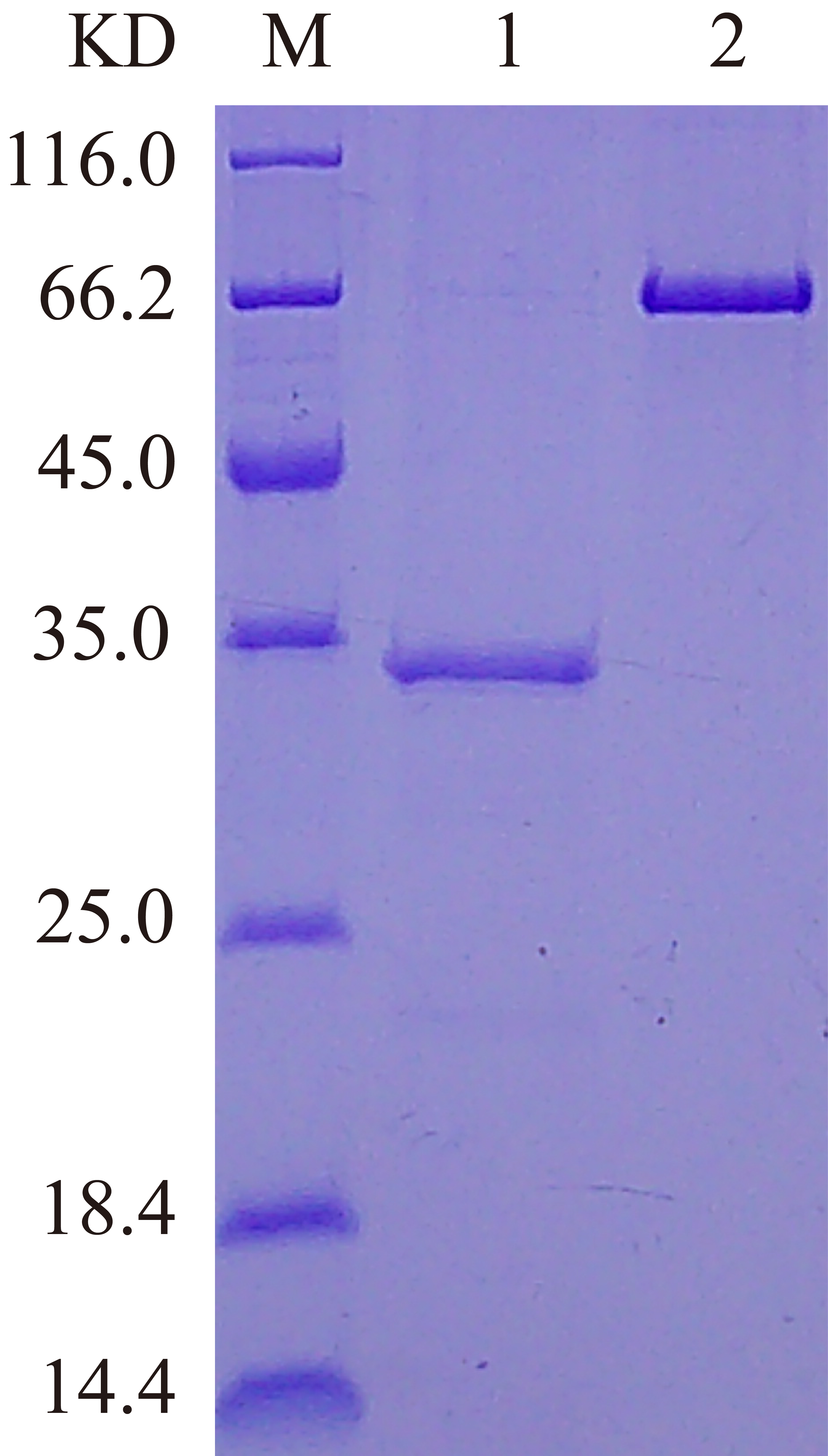

Supplement: Supplementary file 1 [file biomolecules-15-01593-s001.zip › Supplementary File/Figure S8-ú¿Cú⌐.tiff]

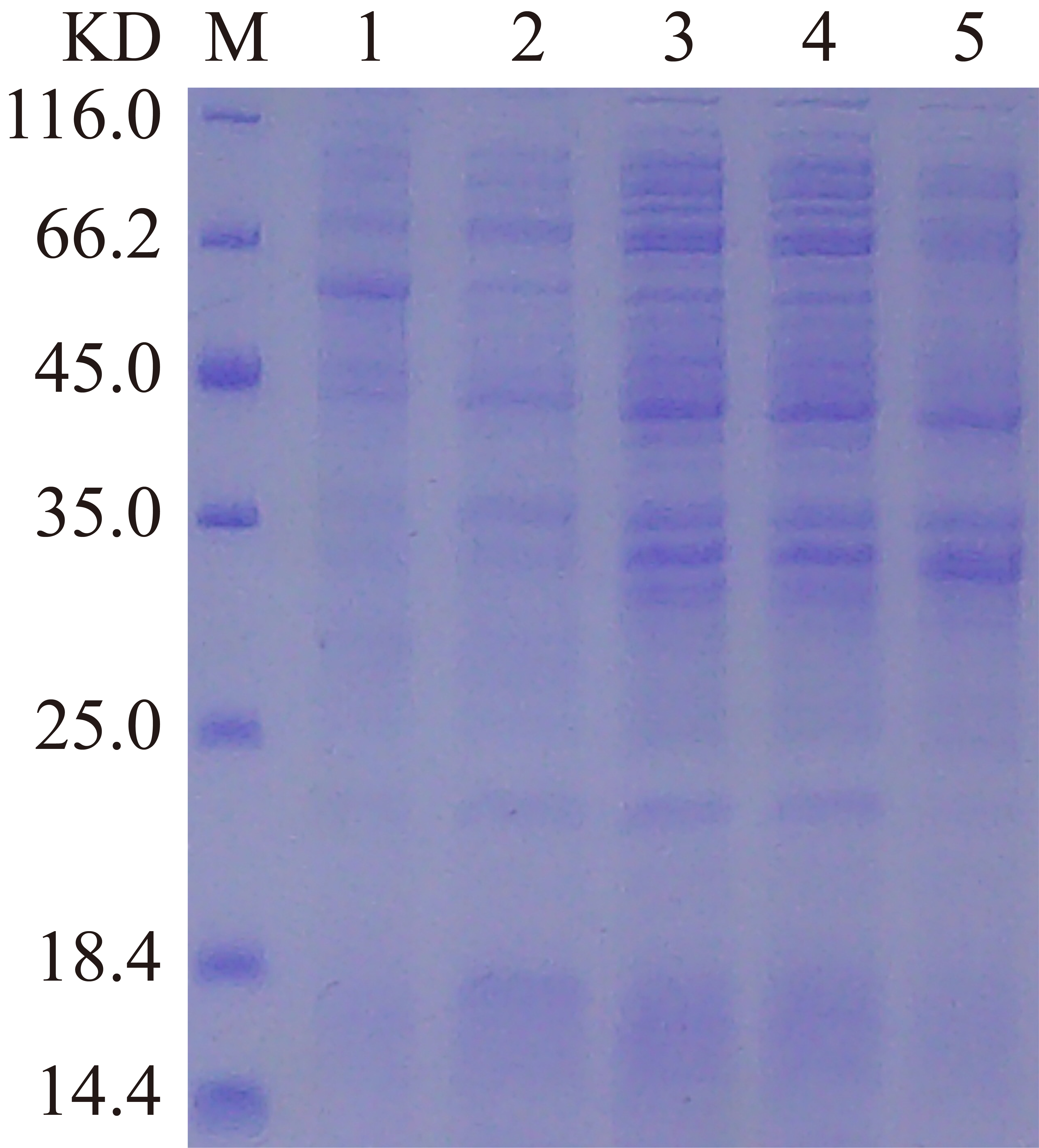

Supplement: Supplementary file 1 [file biomolecules-15-01593-s001.zip › Supplementary File/Figure S9-ú¿Aú⌐.tiff]

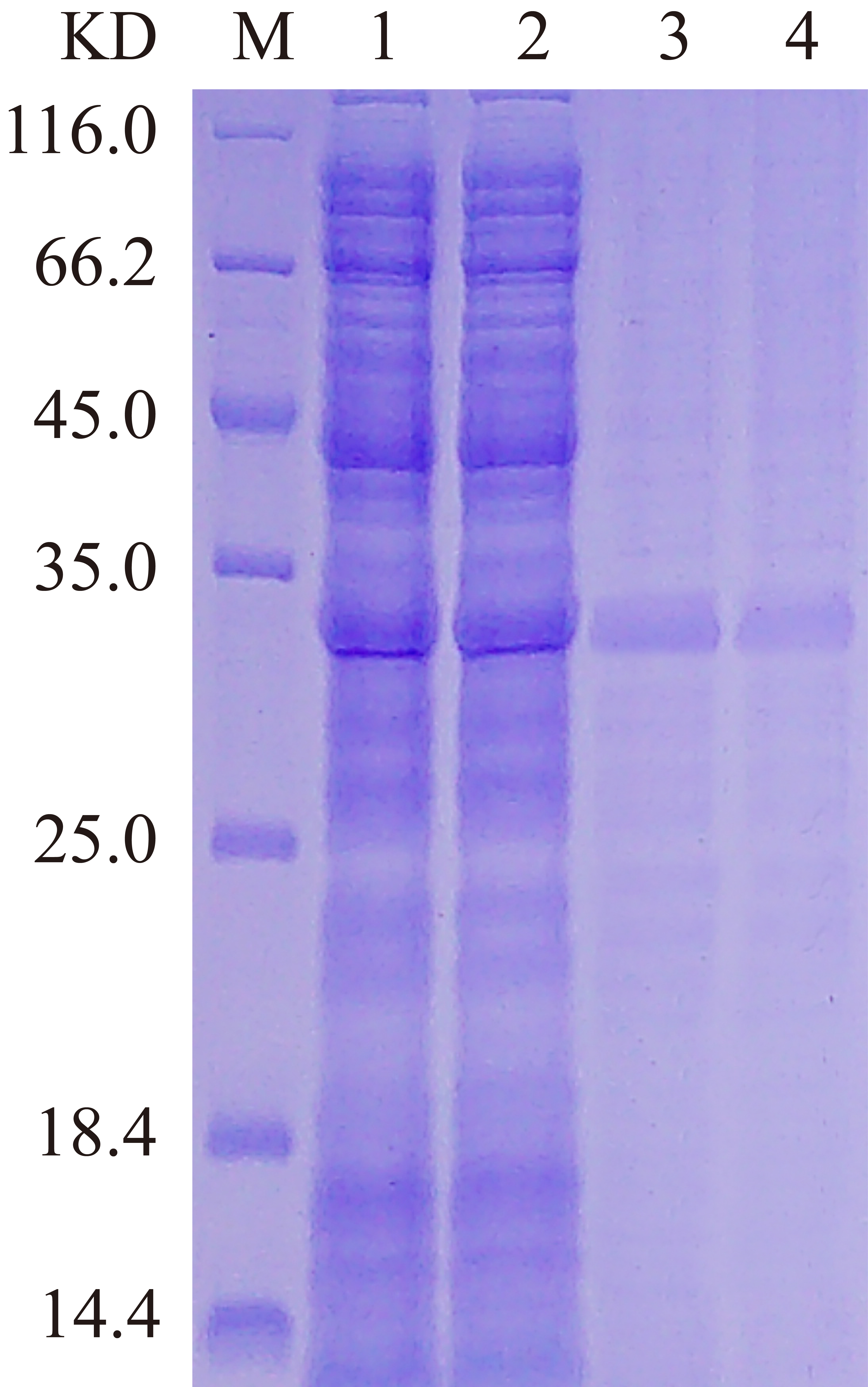

Supplement: Supplementary file 1 [file biomolecules-15-01593-s001.zip › Supplementary File/Figure S9-ú¿Bú⌐.tiff]

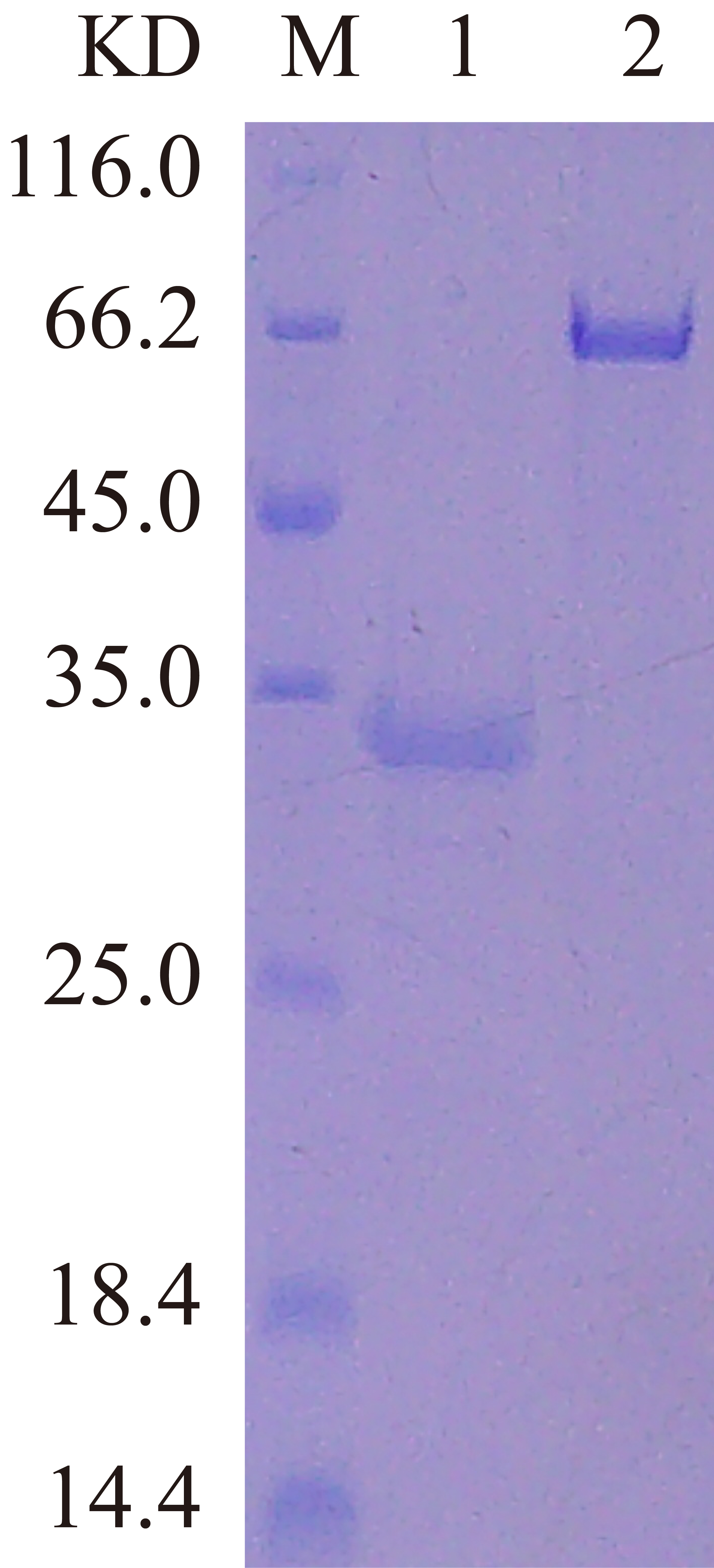

Supplement: Supplementary file 1 [file biomolecules-15-01593-s001.zip › Supplementary File/Figure S9-ú¿Cú⌐.tiff]
